# Small-Molecule Targeting of the Iron-Responsive Element in the APP mRNA 5′-UTR to Control Amyloid Translation in Alzheimer’s Disease

**DOI:** 10.3390/ijms27093978

**Published:** 2026-04-29

**Authors:** Mateen A. Khan, Hassan S. Shaibah

**Affiliations:** 1 Department of Life Science, College of Science and General Studies, Alfaisal University, Riyadh 11533, Saudi Arabia; 2 Department of Anatomy, College of Medicine, Alfaisal University, Riyadh 11533, Saudi Arabia; hshaibah@alfaisal.edu

**Keywords:** Alzheimer’s disease, amyloid precursor protein, structured mRNA, small molecule inhibitors, protein synthesis

## Abstract

Amyloid-β (Aβ) protein, a cleavage product of the amyloid precursor protein (APP), is the main component of neuritic plaques in Alzheimer’s disease (AD), and its accumulation has been considered as the molecular driver of Alzheimer’s pathogenesis. Aβ has been a primary target for therapy since the amyloid cascade theory was put forth, with methods designed to prevent the generation of Aβ. The APP 5′-untranslated region (UTR) mRNA encodes a functional structured iron-responsive element (IRE) that represents a potential target for small molecule inhibitors as an anti-amyloid therapy for AD. Here, we offer a comprehensive strategy that uses RNA-targeted binding to inhibit APP translation. The IRE family is among the few 3-D mRNA regulatory elements with a known 3-D structure. Accordingly, we exploit these structural and functional characteristics as our strategy to target APP IRE structured mRNA to identify anti-amyloid drugs. The mRNA encoding proteins involved in iron metabolism are regulated by this family of similar nucleotide sequences. Post-transcriptional control of cytoplasmic mRNA is a rapidly developing area of biomedicine. Across animals, evolutionarily conserved IRE mRNAs serve as a model system for 3-D mRNAs. IRE mRNAs have shown great promise for chemical manipulation of mRNA and protein expression in biological systems by yielding “proof of principle” data for small molecules targeting mRNA structures. A novel approach to identifying RNA-directed therapeutics to regulate APP expression and Aβ-peptide generation for AD treatments is exemplified by APP 5′-UTR-directed small molecule inhibitors.

## 1. Introduction

Alzheimer’s disease (AD) is characterized by neurofibrillary tangles (NFTs) composed of hyperphosphorylated tau and neuritic plaques composed of misfolded amyloid peptide. Because it generates the amyloid-β (Aβ) peptide [1,2], which misfolds, oligomerizes, and forms fibrils, the protein amyloid precursor protein (APP) is crucial to the pathophysiology of AD. With dementia condition, AD is the most prevalent neurological disease that causes progressive cognitive impairment. The main contributing component to the etiology of AD, according to the amyloid hypothesis, is the buildup of pre-plaque Aβ in the brain. Processing of APP, a crucial protein in AD pathogenesis, is necessary for the production of Aβ [3]. Targeting Aβ has been the primary focus of AD therapeutic development over the last few decades. However, this theory has been seriously called into question by the repeated failures of Aβ-targeted therapeutic trials. In the research and treatment of AD drugs, anti-Aβ therapy is currently a major source of debate. Currently approved small molecule drugs such as acetylcholinesterase inhibitors (AChERs) and N-methyl-D-aspartate (NMDA) receptor antagonists primarily provide symptomatic relief.

Post-translational processing of APP involves both pathological and normal processes and can be either amyloidogenic or non-amyloidogenic [4]. Aβ pathogenicity results from dysregulation of APP processing during the transition from amyloidogenic to non-amyloidogenic pathways. β-secretase and γ-secretase sequentially cleave the APP to generate Aβ. One strategy to lessen the amyloid plaques in the brain is to target the proteolytic enzymes (β- and γ-secretase) involved in APP processing through amyloidogenic. Therapeutic approaches for AD include producing Aβ-specific antibodies, increasing Aβ clearance mechanisms, and triggering autophagy. It has been observed that additional metal ions, particularly Fe^2+^, are crucial in stabilizing and speeding Aβ oligomers. Thus, a possible therapeutic approach to lowering Aβ toxicity is a combination therapy targeting multiple AD pathologies will be more effective than a single therapy, which only addresses one abnormal factor that also targets Fe^2+^ chelators [5].

The amyloid hypothesis has been called into question after decades of drug development research aimed at Aβ resulted in several trial failures [6]. The last five years, however, have been a turning point. A significant advancement in AD treatment is the regulatory approvals and encouraging trial outcomes of multiple target agents to address neurodegenerative diseases. As the first to demonstrate disease-modifying potential, these drugs can remove amyloid from the brain, which gives hope that Aβ will be a promising target for treatment. The fact that no other pathogenic pathway has yet to receive regulatory validation of clinical efficacy makes this a significant milestone in the development of AD drugs [7,8]. Aducanumab, a monoclonal antibody that targets Aβ aggregates, received US Food and Drug Administration (FDA) accelerated approval in 2021 as an Alzheimer’s drug due to its capacity to lower Aβ plaque levels [9]. In 2023, lecanemab, a monoclonal antibody that targets Aβ soluble protofibrils, was approved based on the CLARITY-AD study’s proven clinical effects. The most current monoclonal antibody to be licensed for the treatment of early AD is donanemab, which targets deposited plaques. It was approved in 2024. This series of encouraging outcomes gives new hope and suggests that Aβ-based therapy might be the best course of action.

**Figure 1:**
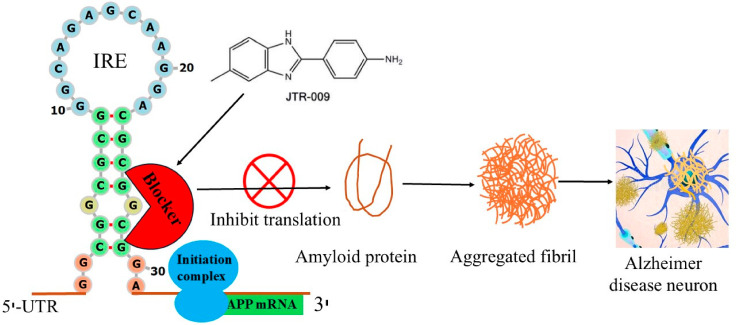
Schematic representation of translation of amyloid protein therapeutic target APP IRE RNA by small molecule inhibitors. Small molecular inhibitors bind to IRE RNA structure and inhibit the synthesis of amyloid proteins through blocking ribosome-initiation factors complex binding.

Figure 1 depicts the expression of the mRNA transcript encoding APP is modulated by the amyloid translation regulation mechanisms of the iron-responsive element (IRE) 5′-untranslated region (UTR). IRP responds to cellular iron levels by either enhancing or repressing translation. Amyloid expression is inhibited when APP IRE is bound and stabilized by IRP at low iron levels. IRP was released from APP IRE at elevated iron levels, which encourages an increase in the synthesis of amyloid proteins. Aβ occurs in several conformations that balance between oligomeric and unstructured soluble monomeric forms under physiological settings. However, as Figure 1 illustrates, excess Aβ synthesis under pathological settings clumps into fibrils, which further causes neuronal death and advances AD. Therefore, using small molecule inhibitors to selectively bind to the structured IRE mRNA and disrupt the first stages of translation regulation of Aβ synthesis could be a promising approach to developing new treatments for AD. This suggested model further illustrates an integrated strategy for choosing a translation blocker, which is a drug-like small molecule that targets APP structured IRE mRNA by direct binding and lowers the expression of Aβ protein in AD patients.

Targeting the coding mRNAs of disease-causing proteins and blocking translation is another promising method to increase protein druggability, especially for overexpressed amyloid proteins [10,11,12]. IRE mRNA in the 5′-UTR, which codes for APP, is specifically targeted by a small molecule such as APP blocker-9, and benzimidazole [13]. In the mRNAs, either before 5′-UTR or after 3′-UTR the coding region, mRNA regulatory structures are typically noncoding. IREs, a collection of similar sequences that are foldable, stem-loop structures that respond to changes in cellular iron level, are the most well-characterized mRNA regulatory structures [14]. Many important proteins in iron homeostasis are encoded by IRE-containing mRNAs. IRPs are protein repressors that specifically identify the IRE structures and block ribosome binding to inhibit protein synthesis [15,16]. In response to elevated iron levels, IRP release from IRE and promoting the pre-initiation complex binding for mRNA translation [17,18].

Protein synthesis rate may be altered by directing small molecules to three-dimensional (3-D) structures in RNA [16,19]. Finding 3-D RNA structures that are functionally relevant and display structures conducive to selective binding in the presence of significant amounts of other nucleic acids is a major difficulty in targeting mRNA [20,21]. Compared to DNA, RNA offers a more cell-specific and diverse range of targets. The potential of RNA targeting is highlighted by the quick development of RNA interference (RNAi) techniques, even though RNAi’s selectivity is dependent on main sequence and secondary structure. The ability to elude biological systems that react to targeting helical RNA structures [22] and the capacity to find potentially helpful RNA targets in genome sequences [23,24] are benefits of employing small molecules to target 3-D RNA structures. Additionally, by employing standard sequencing procedures on small amounts of materials, chemical foot printing techniques which are more successful for nucleic acids than proteins allow for the precise location of small molecule binding to an mRNA target [25]. A drug-like small molecule or RNA-binding molecule that selectively binds to a physiologically significant location in mRNA and alters the rate of encoded protein synthesis can be found using chemical foot printing technique.

This review first provides an overview of recent studies showing the formation of Aβ, emphasizing the function of structural mRNA and the translational regulation of APP mRNA by IRP and Fe^2+^. We provide an overview of the research and clinical results of the anti-Aβ small molecule target. Further an update on therapeutics, which include small molecules that either directly or indirectly interfere with inhibition of Aβ production are briefly explained. Finally, we discuss future perspective, including multi-target approaches and early prevention strategies.

## 2. Aβ Generation and Plaque Formation

The main factor contributing to the development of AD is Aβ. APP, which belongs to a conserved protein family that includes APP-like proteins, is the source of Aβ [26,27]. The single-pass structure of APP, a transmembrane protein that is extensively distributed throughout the body, consists of an intracellular domain, a hydrophobic transmembrane domain, and an extracellular domain. Proteolytic enzymes β-secretase and γ-secretase sequentially cleave APP in the amyloidogenic pathway, which results in the production of Aβ [28]. APP is usually cleaved at the α-secretase site at amino acid seventeen of the 40–42 amino acid Aβ domain, which is the non-amyloidogenic pathway. These cause APP ectodomain to be cleaved, forming the APPsα fragment and a C-terminal fragment (CTF-α) [29]. Because the cleavage site of APP is within the Aβ domain, the cleavage of APP by α-secretase inhibits the formation of Aβ. Short p3 peptide and APP intracellular domain (AICD) are then released when γ-secretase cleaves CTF-α [30]. β-secretase is a membrane-bound aspartyl protease enzyme that can initially cleave the precursor protein at the beginning of the Aβ domain, resulting in a C-terminal fragment of CTF-β and sAPPβ. β-processing is the initial stage of the APP amyloidogenic pathway employing β-secretase, which creates Aβ [31]. Extracellular Aβ peptide fragments and AICD are produced when γ-secretases further cleave CTF-β [32]. In APP metabolism, one important β-secretase is the β-APP cleaving enzyme (BACE1). In AD patients, higher levels of BACE1 protein and activity have been seen in specific brain areas [33]. Several small compounds function as BACE1 inhibitors; some of these therapeutic candidates are presently undergoing clinical studies [31]. Because of their crucial roles in the production of Aβ, BACE1, and γ-secretase are potential therapeutic targets for treatment in AD.

Following secretion, Aβ aggregates into various soluble species, which then transform into cross-β-sheet fibrils to produce plaques. Aβ_40_ makes up the majority of Aβ in a healthy human brain, while excess Aβ_42_ is created in pathological conditions and mostly aggregates as amyloid plaques. When compared to Aβ_40_, Aβ_42_ exhibits faster aggregation kinetics and severe neurotoxicity [34]. Additionally, an off-pathway self-association of a native unfolded Aβ peptide can result in stable oligomeric aggregates [35]. The oligomers, which are thought to occur in the lag phase of fibril production, have been demonstrated to be the most toxic type of Aβ [36]. In the brain, these oligomers can serve as nucleation sites for the creation of additional oligomers and higher-ordered aggregates. Metal ion (Fe^2+^) coordination with Aβ has been shown in numerous investigations to result in increased aggregation rates, oligomeric state stabilization, and reactive oxygen species (ROS) production [37]. Other research has demonstrated that iron also binds to amyloid in vivo; iron is bound to plaques in the central nervous system (CNS) tissue of both AD patients and animal models [38].

Aβ is a major cleavage product of the APP, is enhanced by iron, as found in the amyloid plaques of AD patients. In addition to Aβ, iron can also be bound by tau, become interact in microglia [39,40]. Iron is present from the diffuse regions to the dense core of amyloid plaques. A large portion of this iron is firmly bound; for example, it can be released after CNS tissue is treated with proteinase K and/or detergents [38]. Iron probably binds Aβ before it aggregates, rather than plaques forming first and then iron binding. Iron may serve as a catalyst to encourage Aβ aggregation and fibril production [41,42]. Therefore, iron can promote the aggregation and formation of plaques if Aβ is not cleared in a timely or efficient manner, for instance because of decreased expression of lipoprotein receptor-related protein 1 (LRP1) with aging or decreased clearance of Aβ/ApoE4 [43,44]. In addition to Aβ removal of labile iron from the interstitial fluid, iron that is firmly bonded inside the plaques may also be a mechanism that reduces the amount of iron available for organelle function. Although it is unclear if iron can act as a catalyst when bound to Aβ [45], it is possible that iron is redox-reactive, which could cause tissue damage at and around vessels [46,47]. However, heme appears to be redox reactive after it binds Aβ [48,49].

Iron tightly regulates the expression of the Alzheimer’s APP gene at the level of APP 5′-UTR mRNA translation, similar to iron regulates the translation of ferritin IRE mRNAs in their 5′-UTRs. The overexpression of amyloid, its aggregation, the death of neurons, and the progression of AD are all caused by anomalies in the IRP/IRE signaling pathway brought on by iron dysregulation in the brain tissues. The homoeostasis of APP can be affected by variations in the quantities of iron within cells, which can affect IRPs and ribosome attachment to the IRE mRNA [50]. Among its non-pathogenic functions, APP regulates intracellular iron homeostasis. The iron transporter ferroportin (Fpn) is bound by APP, which controls the quantity of iron that leaves cells [51]. Additionally, iron binds to IRPs to regulate the synthesis of proteins with the IRE sequence [18]. The iron-binding proteins are comparable to the IRE domain of the APP transcript [52]. Consequently, APP can regulate the amounts of iron in neurons, which affects the synthesis of APP.

Neuronal degeneration and mortality are linked to these fibrils spreading throughout neurons, neuro-fibrillations, and the formation of insoluble Aβ plaque [53,54]. One of the main causes of Aβ fibrillization is its concentration, which can be caused by somatically driven non-disjunction events or genetically inherited over-expression of the APP gene [55,56,57]. Therefore, lowering APP protein levels may be a way to change the condition. However, Aβ protein is thought to be undruggable due to its inherent disorder and lack of pockets that can normally be bound by small molecule inhibitors.

## 3. APP mRNA Encodes Functional 5′-UTR IREs

A functional IRE stem loop with a distinct CAGA box is encoded in the 5′-UTR of the APP transcript. IREs are made of simple RNA hairpins. IRPs’ differential recognition of IREs regulates the translation of mRNAs. The 5′ or 3′-UTRs of mRNAs that encode iron metabolism proteins contain about thirty nucleotide sequences known as IREs. Every member of the IREs family possesses two types of conserved information: information unique to the IRE mRNA and information shared by all IRE mRNAs. The interleukin-1 (IL-1) response acute box domain is directly upstream of the IRE in APP mRNA [58]. The UTRs of various mRNAs that generate proteins involved in iron metabolism contain the IRE. The most common RNA elements, hairpins, are useful for binding regulatory proteins [59,60,61].

**Figure 2:**
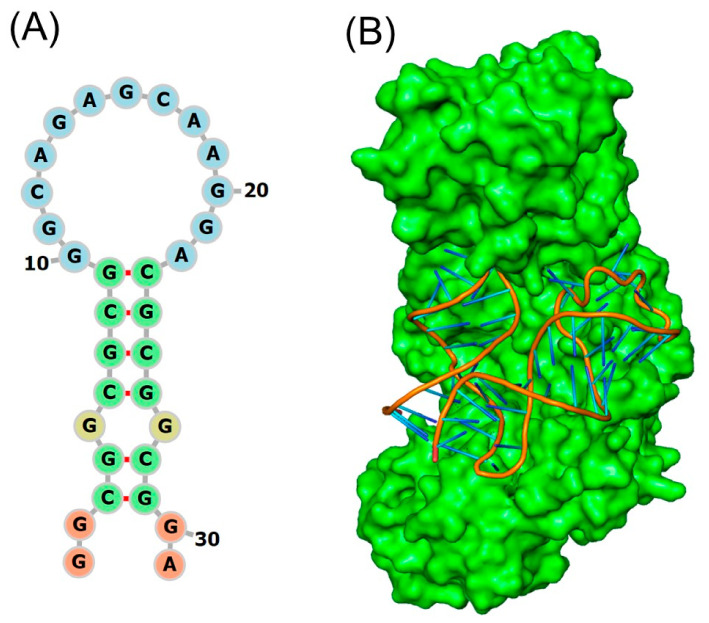
Structural models of APP IRE RNA and IRP1 complex with APP IRE RNA. (**A**) APP IRE secondary structural model and (**B**) APP IRE in complex with IRP1 protein. Modification of figure with permission originally published in refs. [66,80].

Figure 2A shows the secondary structure predictions that the IRE can be folded into a stem-loop structure with flanking sequences of variable length and sequence that lengthen the stem. With a conserved six-nucleotide loop on top and two short helices divided by a protruding C, they are folded into a hairpin. RNA secondary structure is dominated by base paired stems and hairpin loops. All IRE mRNAs have a short (9–10 bp) double-stranded helix with an unpaired C at the core that results in a bulge. The variations in IRE sequences among different mRNAs are relatively small because all IREs form the mRNA A-helix with the same bulge C and terminal loop sequence [62]. Base pairing in the APP IRE may produce a crucial AGA tri-loop [63]. Together with the three nucleotides that lie between bases 14 and 18, a special cross-loop base pair is responsible for the tri-loop look (15–17). The trailing 3′-base at position nineteen is thus left hanging by itself. A pseudo-tri-loop motif is the collective term for nucleotides 14–19 [64]. In contrast to the C8 residue, the G7 residue is anticipated to be a bulge base in the APP IRE predicted structure [63]. A functional IRE is also encoded in the APP transcripts [65]; the canonical loop helix of the ferritin and APP IRE stem loop are quite similar [65]. Ferritin IRE/IRP1 and the APP IRE/IRP1 complex [66] appear to have similar binding stabilities. Many additional proteins, such as APP in AD [63,65], α-synuclein in Parkinson disease [67], and α-hemoglobin stabilizing protein [68] have been shown to include IRE-containing mRNAs. Thus, change in cellular iron levels can deregulate IRP1 binding to IRE RNA stem-loops, affecting APP translation. The IRE RNA stem-loop in APP mRNA is the focus of current efforts in RNA based anti-amyloid therapy. This IRE confers iron-responsive translation of APP, and its unique RNA structure is a drug target to suppress APP expression.

APP IRE binds to IRP1 in an analogous manner to ferritin IRE. APP IRE bound IRP1 in a cleft using the functionally active residues of the IRP1 binding site. Because of its structural complementarity, APP IRE was able to securely fit into the binding pocket of the IRP1 [66]. The APP IRE in the IRE/IRP complex flips out its terminal loop bases, and the IRE backbone is deformed by an abrupt mid-helix turn. The secondary structure of APP IRE mRNA is predicted to contain both the conserved C8 residue and the pseudo-tri-loop [63,66]. The in vivo response to iron levels, when there is at least an order of magnitude difference in APP and ferritin mRNA, exhibits the greatest fluctuations in IRP1 binding in solution [66,69]. To keep IRE regulation functional, it is essential to preserve the high affinity binding of IRE RNA with regulatory protein, IRP1. IRP1 recognizes a three-dimensional (3-D) RNA structure rather than a two-dimensional (2-D) RNA structure.

Figure 2B depicted the specific binding model of the APP IRE in complex with IRP1 protein. IRE is bent, and IRP1 protein has an L-shape. In the complex, the IRE RNA is inserted between protein domains 1-2 and 4. APP IRE interacts with a set of functionally active eleven amino acid residues of the IRP1 binding site. IRP1 is depicted as a cartoon model (pale green), and APP IRE is shown in light brown in an element, ball, and stick model. A large RNA surface remains exposed, inviting RNA interactions with other molecules and metal ions even while bonded to IRP1. Binding specificity in the IRP1/APP IRE RNA complex is achieved with only two widely separated contact sites [62,66]. At the apex of the IRE pseudo-tri-loop motif, several nucleotide base viz., A15, G16, and U17, are involved in hydrogen bonding with amino acid residues of IRP1. These interactions are necessary for effective RNA/protein regulation. Consequently, base pairs 15-16-17 ought to feature an AGU pseudo-tri-loop for an IRE. If this apical loop is not A16G16U17, a weaker IRE/IRP contact may occur, but in vivo regulatory function may not be supported. The structure and sequence of individual rings of IREs, which are usually made up of an apical loop motif and a stem-loop element that are divided from a lower stem by a C-bulge, are highly conserved in mammals [70,71,72,73]. Many IRE-like structures have now been identified in various mRNAs, some of which encode proteins crucial for iron homeostasis and neurodegeneration [74].

Base pair variations in the IRE helix base pairs of the 5′-UTRs and sequence alignment of APP mRNA bulges compared to ferritin [18] and mitochondrial aconitase IRE mRNA transcripts. The APP IRE, which is different from the well-known ferritin IRE but encodes a sequence similar to the IRE, is one example of this homology [75]. Variations in the quantities of proteins encoded in mRNAs within cells can be attributed to varying mRNA stabilities [14]. Members of the IRE mRNA family exhibit quantitative difference in binding of IRP repressor, eukaryotic initiation factors (eIF4F) activator, or even the Fe^2+^ signal itself due to mRNA bulges and base pair differences in mRNA. Diverse in vivo iron responses are caused by diverse IRE structures. In vivo, ferritin protein synthesis was enhanced more than mitochondrial aconitase by the same concentration of Fe^2+^ in the same tissue, such as the liver. This phenomenon is explained by slight differences in the structure of different IRE mRNAs. It has previously been shown how the IRE-like mRNA stem loop locations are arranged in APP, ferritin, and mitochondrial aconitase [76]. The distance between the IRE, mRNA cap, and AUG start point at the 5′-UTR can influence the effectiveness of regulation [18]. Depending on the type of gene, the distances between IRE to start and IRE to cap can change. However, the conservation of a single IRE, like APP IRE, is consistent across species, ferritin IRE differs significantly less from IRE sequence changes in other mRNAs within the same species [77].

The modulation of ferritin mRNA translation through an IRE in their 5′-UTRs is comparable to how iron has been shown to affect the expression of Alzheimer’s APP. IRPs bind to IRE mRNAs and control ferritin and APP post-transcriptionally. Ferritin and APP 5′-UTR activity is controlled by iron-sensing IRP/IRE signaling pathways, which cause protein biosynthesis. Neurotoxic oxidative stress resulted from the uncontrolled accumulation of redox-active Fe^2+^ following the loss of the protective APP and ferritin axis. Ferritin, APP, and iron-storage proteins, which help maintain membrane-bound Fpn and reduce intracellular levels of toxic redox-active Fe^2+^, have recently been shown to increase in concentration and exhibit time-dependent activation in neuronal cells treated with ferric ammonium citrate [78]. By regulating the translation of ferritin mRNAs, IRPs are known to modify intracellular iron homeostasis. APP is a metalloprotein expressed to detoxify metal-controlled oxidative stress is undoubtedly supported genetically by APP mRNA translational control by iron and APP gene transcriptional regulation by copper [79]. Finding new therapeutic approaches that can maintain the iron and APP in the cell will be aided by focusing on the APP 5′-UTR mRNA transcript.

## 4. APP mRNA 5′-UTR Translation by IRP1

5′-UTR of APP contains a functional and conserved IRE that binds to IRPs and mediates translational repression of APP mRNA. The IRE in the 5′ UTR of APP shares homology with the IRE in the mRNA transcript for the iron storage protein ferritin and is responsive to IRP1 [63]. In addition to iron, the UTR upstream of the IRE can accelerate translation of ferritin and APP in response to IL-1 responsive acute box element [81,82]. Several RNA-protein complexes are produced by subtle, conserved modifications in the IRE structure and sequencing. The stability of RNA-protein complex binding varies slightly among members of the IRE family. There are physiological implications to the changes in IRE/IRP stability. For instance, ferritin and mitochondrial aconitase IRE/IRP are ten times less stable under the same circumstances, whereas the stability of IRE/IRP binding in a solution differed by two times between APP and ferritin [66]. Changes in IRE stabilities lead to changes in the amounts of cellular proteins encoded by mRNAs. Ferritin IRE and IRP form a much more stable complex than APP and mitochondrial aconitase IREs. Because of this, ferritin mRNA translation is more resilient to slight changes in intracellular iron levels than the other mRNAs, which together make up the less stable IRE/IRP complex. From a physiological point of view, the structural distinctions between ferritin, mitochondrial aconitase, and APP IRE correlate to functional differences in each mRNA-encoded protein’s cell metabolism. This is due to the fact that while ferritin and APP are periodically required for their iron-concentrating functions, mitochondrial aconitase is constantly required for bioenergetic cellular activity.

The expression of APP and its proteolytic products is influenced by cellular iron levels. With increasing iron levels, both the translation of APP and the levels of its proteolytically cleaved components (Aβ42, C83, and C99) increased in ARPE-19 cells, a cultivated retinal pigment epithelial cell line [83]. Ferric ammonium citrate enhanced APP synthesis, β-secretase activity, and Aβ42 levels in the neuroblastoma cell line SH-SY5Y [84]. Ferric chloride increased APP and Aβ42 levels in BV-2 cells and the microglial cell line [85]. Moderate iron levels (by hemin) enhanced soluble APP levels in HEK 293 cells (human embryonic kidney cells transfected with APP), but not proteolytic fragments [86]. Iron restriction by chelation decreased the amount of iron in the brain and the levels of APP and secretase enzymes in normal mice [87]. Together, these findings show that the concentration of iron affects the synthesis of APP and Aβ. Several reports demonstrated the specific interaction between IRP1 and APP IRE [63,66]. Furthermore, iron may enhance secretase activity to generate proteolytically cleaved products [84]. Together, these findings imply that IRP1 stays bound to the APP IRE to suppress APP translation under low iron conditions, increased cellular iron levels promote the synthesis of APP and its cleavage products [88].

The APP IRE stem loop preferentially binds with IRP1 with high nanomolar affinity, similar to ferritin IRE [66]. This strong APP IRE/IRP relationship in response to intracellular iron levels was similarly apparent in SH-SY5Y cells, H4 neuroglioma cells, and human patients brain lysates [63]. The study of IRP1 interaction with APP mRNA in the human brain confirmed this finding [63]. It has been proposed that the altered binding of IRP1 to the ferritin IRE reflects an unidentified pathogenic feature of the AD brain. In order to preserve brain neurons recovering from ischemic stroke and bleeding, IRP1 may play a significant role in controlling APP translation [89]. The important discovery that APP plays a crucial role in Fpn-dependent iron export and neuronal iron inflow can be connected to the acute protective up-regulation of APP in certain brain regions [90]. In response to cellular iron levels, iron-induced expression of APP is regulated at the translational level by mechanisms that are similar to but different from those controlling ferritin translation [63].

Iron overload contributes to the development of AD by inducing Aβ accumulation, NFT production, and overexpression of APP. Fe^2+^ homeostasis and the genetics of AD have been directly linked through the presence of an IRE in the 5′-UTR of the APP mRNA transcript [18,65]. IRE is selectively responsive to intracellular iron levels in a way that controls the iron-dependent regulation of intracellular APP synthesis. It has been shown that Fe^2+^ levels affect APP mRNA translation in astrocytes and neuroblastoma cells by a mechanism similar to iron modulation of ferritin mRNA translation [65,81]. The cellular iron balance is altered at the translational level via the signaling cascade of IRPs and IREs [91]. Due to the two separate locations of IRE in mRNA, iron can have opposite effects on different IRE RNAs. When there are IREs in the 5′-end control mRNA translation through ribosome-initiation factor complex binding. On the other hand, the IREs in the 3′-end control turnover (degradation) [77,92].

**Figure 3:**
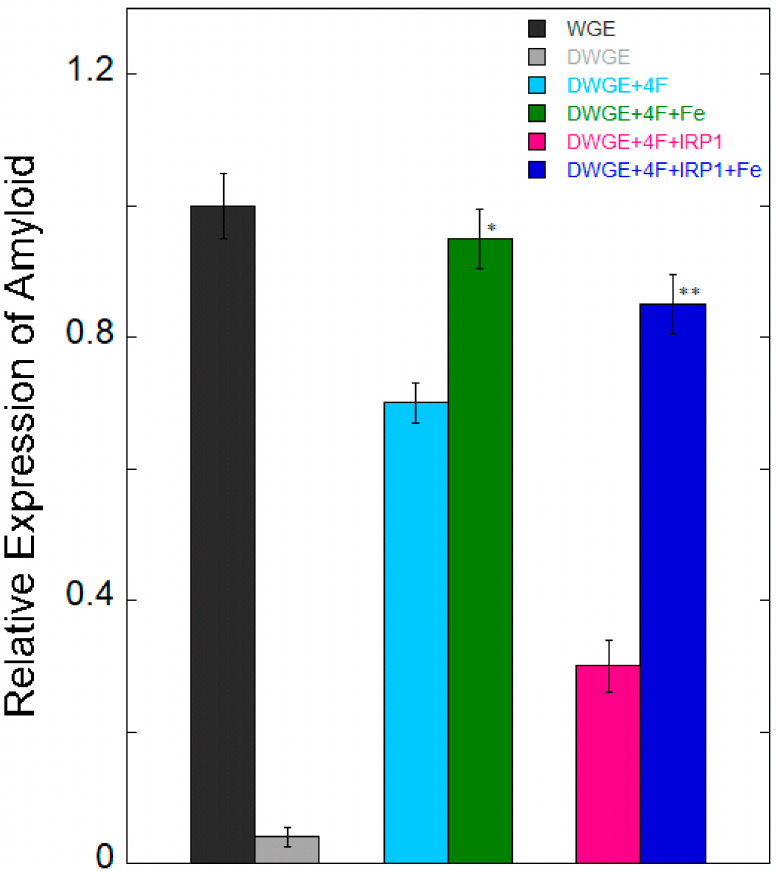
Fe^2+^ enhances eIF4F-dependent protein synthesis, directed by APP IRE mRNA. IRP1 inhibits the translation of APP mRNA. eIF4F-depleted wheat germ extract (WGE) supplemented with eIF4F and IRP1, confirming the contribution of eIF4F and IRP1 to mRNA for protein synthesis. This figure was amended with permission from ref. [88]. Error bars point to the standard deviation. Significant different from eIF4F + RNA (* *p* < 0.02) and eIF4F + IRP1 + RNA (** *p* < 0.01) with Fe.

Figure 3 showed the rescue of APP mRNA translation by exogenous eIF4F in eIF4F-depleted cell extracts and recapitulation of iron enhancement to relate the binding studies of APP IRE and IRP1 with translational studies of cultured cells and animals. The importance of eIF4F binding to APP IRE for protein synthesis was confirmed when exogenous eIF4F was added to initiation factor depleted cell extract to restore APP synthesis. When Fe^2+^ was added to depleted cell extracts supplemented with exogenous eIF4F, it further increased the translation of APP mRNA, indicating the role of Fe^2+^ in the translation regulation of APP. However, the translation of APP is inhibited by the presence of IRP. Additionally, adding Fe^2+^ reversed the inhibition of protein synthesis caused by IRP, which is consistent with the influence on the stability of IRP binding [66] and the activation of APP synthesis in living cells and animals [63]. These findings show that Fe^2+^ promotes initiation factors binding to reversed IRP translation regulation and causes IRP to be released from APP mRNA. Two regulatory proteins, repressor (IRP1) and activator (eIF4F) can be competitively bound to the APP mRNA, demonstrating the significant role that untranslated mRNA of the 5′-UTR can have on the expression of genes and the protein synthesis rates. These proteins respond to the cellular iron levels by mediating protein synthesis. At high iron level, iron removes IRP1 from the APP RNA/IRP1 complex, whereas promotes eIF4F binding to APP mRNA, thus enhancing the expression of the amyloid protein. It is known that IRP binding to 5′-UTR limits ribosome binding and mRNA translation, however it is yet unclear which precise steps in the sequence of events in releasing IRP and assembling an initiation factors complex with an IRE-mRNA. IRP binding to an IRE at the 3′-UTR of transcripts can protect target mRNA in iron-deficient cells from endonuclease cleavage [92]. IRPs and IRE can thereby enhance target mRNA translation and extend transcript half-lives. On the other hand, target transcripts in iron-depleted cells are less likely to be translated due to endonuclease activity and degradation caused by IRP separation from an IRE at the 3′-UTR. The IRP/IRE interaction can both disrupt and increase ferritin transcript translation and destabilize transferrin receptor (TfR) mRNA during iron overload [92]. Iron export and storage may therefore be increased while iron absorption is limited in situations of iron overload [93]. Until recently, it was unknown how cellular iron signals affect IRP affinity for APP IRE [66].

Moreover, the complex process of ribosomes bind to multiple initiation factors and proteins to create pre-initiation complex that consists of initiator tRNA, mRNA, ribosomal subunits, and approximately eleven translation initiation factors. Several features suggest that IRE structures cooperate with other elements of the mRNA structure since there is little distance between the start of the mRNA and the IRE [18]. Certain mRNAs, like mitochondrial aconitase, have the initiator AUG embedded in their IRE; the functional significance of initiation at the IRE is unknown. These IREs structures selectively bind iron to regulate the stability of a protein repressor complex that inhibits ribosome binding and protein synthesis. Up-regulation is the outcome of activator protein (eIF4F) binding to IRE. The IRE mRNA is characterized by the common loop and bulge that make up the unique protein binding sites for each IRE. The binding of metabolites (Fe^2+^) and repressor proteins (IRPs) is altered by base pair changes in the individual IRE helix, which converts protein synthesis in vivo environmental iron. A regulatory feedback loop with iron is created when the iron signal is consumed by ferritin protein expression. As a result, ferritin protein lowers the rates of ferritin protein synthesis, increases the binding of IRP1 to ferritin IRE mRNA, and decreases the quantity of free cellular iron.

Through Fe^2+^ sensing, IRPs may both support and inhibit translation control in the IRE/IRP system [77,92]. In vivo, these systems regulate iron transport, storage, and regulation at the cellular and/or systemic levels via controlling iron homeostasis. However, when the iron regulating system is out of balance as a result of genetic abnormalities and/or illnesses affecting iron balancing proteins, an excess of iron in the brain tissues results in neuronal death [94]. Increasing the amount of iron in cells can change both the protein itself and the structure of IRE mRNA, which influences the protein interactions. Disruption of the IRP/IRE signaling system will impair iron homeostasis, which may contribute to the overexpression of amyloid protein and the advancement of AD pathogenesis.

## 5. Small Molecule Therapeutic Strategies for AD

### 5.1. Secretase Inhibitors Therapy

Secretase inhibitors have been extensively studied as a potential therapeutic approach for AD because of their ability to inhibit Aβ production. However, clinical trials of secretase inhibitors have often failed despite strong preclinical efficacy. Specifically, the most studied small molecule approach for AD, β-secretase inhibitors, has repeatedly failed in clinical development. Despite successfully lowering cerebral fluid and plasma Aβ levels, BACE1 inhibitors (such as atabecestat, umibecestat, lanabecestat, elenbestat, and elubecestat) have not yet received final approval [95]. These failures highlight the difficulty of long-term BACE1 suppression because the enzyme has a variety of physiological substrates outside of APP, such as involvement in myelination and synaptic function, which can result in toxicities based on mechanisms [96]. The first small molecule BACE1 inhibitor with oral availability and blood-brain barrier permeability is verubecestat [97]. It progressed to phase 3 trials in patients with mild to moderate AD, but it was ineffective and even worsened cognitive function [96]. In general, the lack of therapeutic benefits and toxicity have been the main reasons for BACE1 inhibitor studies to fail. Therefore, none of the BACE1 targeting strategies have yet to show disease-modifying efficacy, even though small molecules have helped manage AD symptoms.

Furthermore, because they can prevent the generation of Aβ, γ-secretase inhibitors have also been studied as possible treatment targets for AD [98]. Early investigations showed that semagacestat, one of the first γ-secretase inhibitors to enter late-stage trials, decreased plasma, and CSF Aβ levels. However, because there was no clinical benefit and it was linked to intolerable toxicities, the phase 3 study was stopped [99]. The limitations of wide γ-secretase inhibition were brought to light by these failures, especially the off-target effects resulting from interference with Notch signaling. As a result, scientists have worked to create a far more targeted γ-secretase inhibitor that exclusively interferes with the synthesis of Aβ. In animal models, avagacestat, a newly discovered arylsulfonamide γ-secretase inhibitor with strong selectivity for APP over Notch, successfully decreased the level of CSF Aβ without causing any harm due to Notch. Phase 2 trials were stopped because of gastrointestinal and dermatological adverse effects, despite the fact that it was once thought to be a promising treatment with the capacity to selectively inhibit APP processing [100].

Due in large part to the toxicity caused by the absence of substrate-specific inhibition, clinical studies employing β- and γ-secretase inhibitors have failed. The focus has switched from non-selective inhibition to APP processing to drugs that stabilize Aβ, modify its receptor connections, or improve cellular resilience. Nevertheless, efforts to identify small molecules to suppress APP and Aβ generation.

### 5.2. Monoclonal Antibodies Therapy

This strategy aims to design monoclonal antibodies or synthetic peptides to decrease Aβ and slow the AD progress. Immunotherapy is regarded as one of the most promising approaches to AD modification. monoclonal antibodies that lower brain Aβ by attaching to it and delaying the progression of the disease. Although they targeted soluble monomeric Aβ, early generation monoclonal antibodies were unable to impede clinical efficacy. The potential to eliminate plaque and somewhat reduce cognitive decline has been shown by more recent second-generation monoclonal antibodies that target aggregated forms of Aβ [101]. First generation monoclonal antibodies includes bapineuzumab [102], solaneuzumab [103], and crenezumab [104]. Unfortunately, the accumulation of amyloid was not stopped by these antibodies. Instead of targeting monomers, second generation monoclonal antibodies, including lecanemab, aducanumab, gantenerumab, and donanemab, were made to target aggregated pathogenic Aβ [105]. Amyloid-related imaging abnormalities (ARIA) are often linked to anti-Aβ antibody therapy. ARIA may also result in brain atrophy, which would indicate that the ventricles are larger.

The first AD treatment authorized by the FDA is aducanumab. This particular human monoclonal antibody binds Aβ aggregates with selectivity [106]. Imaging studies have demonstrated that it reduces plaques [107]. Since the FDA did not mandate phase 2 trials, Biogen did not carry them out, which drew criticism from certain specialists who felt that there was insufficient evidence to establish its effectiveness [108]. Biogen started two crucial phase 3 clinical trials, EMERGE and ENGAGE, based on their encouraging outcomes. The purpose of these trials was to assess aducanumab’s capacity to maintain cognitive function in individuals with early-stage AD [109]. Despite conflicting findings, the high dosage subgroup aducanumab treatment improved cognitive deficit in the participants. Aducanumab was approved by the FDA in June 2021 for medical use. It was considered controversial due to the lack of sufficient evidence to support its efficacy [110]. It was taken off the market due to ongoing debate about its safety and effectiveness. Although its withdrawal highlights implementation issues, its approval is nevertheless a significant milestone that shows that Aβ immunotherapy can modify the pathology in AD.

Lecanemab is an IgG1 monoclonal antibody that specifically binds to large, soluble Aβ protofibrils. It is produced from a monoclonal antibody mouse. Phase 1 trials have demonstrated antibody safety and lack of significant side effects [111]. It was found to effectively lower brain amyloid and improve cognitive decline in the highest dose group during the phase 2 trial [112]. To ascertain lecanemab therapeutic effectiveness in treating patients with mild cognitive impairment or early AD, the phase 3 Clarity AD trial was initiated in March 2019. Amyloid levels in the brain were decreased by lecanemab therapy, which also lessened cognitive and functional impairment. The FDA decision to convert lecanemab to conventional approval was backed by these data [113]. It proved to be clinically beneficial, and in 2023 it was granted FDA conventional approval, making it the first fully licensed amyloid-directed medicine. The amyloid theory was strongly supported by lecanemab success, but its effectiveness requires patients to be in the initial stages of the disease.

Donanemab is a humanized IgG1 monoclonal antibody that targets and eliminates existing amyloid plaques from the brain [114]. Early findings from phase 2 clinical trials provided compelling evidence that donanemab could slow down the tau and amyloid burden. Consequently, the FDA approved donanemab for early symptomatic AD in July 2024 [115,116].

Gantenerumab is a human monoclonal antibody designed to target Aβ fibrils by identifying a structural epitope on Aβ aggregates [117]. Amyloid burdens were decreased and CSF Aβ41 levels returned to normal after gantenerumab treatment. However, gantenerumab did not show a meaningful therapeutic benefit, according to cognitive data. A new version of gantenerumab formulation exists known as trontinemab, which has a Fab fragment to improve penetration to the blood–brain barrier (BBB). Trontinemab entered the brain fifty times more than unmodified gantenerumab bound to Aβ plaques.

Due to the long preclinical course and slow onset of AD, when pathology develops years before symptoms appear, significant neurodegeneration is frequently evident at diagnosis. Recent anti-Aβ monoclonal antibodies like aducanumab, lecanemab, and donanemab have demonstrated that early AD can be slowed by plaque clearance, especially in patients with moderate dementia or cognitive impairment. Due to their class-specific risks of ARIA and IV infusion requirements, these medicines necessitate MRI monitoring. The majority of authorized anti-Aβ monoclonal antibodies prefer fibrillar plaque formations, while soluble Aβ oligomers, which are thought to be the more synaptotoxic and diffusible species, are less successfully neutralized. This could potentially restrict the amount of cognitive gain that clinical studies can obtain [118]. They are the first disease-modifying treatments to change the course of early AD, and further research aims to increase efficacy and safety; even though clinical benefit is still limited and accessibility is limited by them being expensive, time-consuming, and unpredictable, these treatments are still used in the development of current formulations [119].

It is noteworthy that there is little correlation between the amount of amyloid deposits in the brain and the patient’s lifetime level of cognitive impairment. Some claim that the presence of Aβ is only a marker for the existence of AD and not the primary cause of pathophysiology because of this weak correlation. We observe a wide variety of responses in most AD clinical trials; some individuals exhibit no clinically significant response at all, while others exhibit significant responses. In developing new drugs, it is crucial to identify responder characteristics from these studies. According to recent research, once AD is diagnosed, therapy will inevitably fail because neurodegeneration has already begun. Amyloid-reducing medications may be more effective if treatment may be started before the clinical dementia symptoms manifest. However, the lack of correlation between pharmacological effects in clinical trials should alert us to the possibility that there may be more pathways leading to the final clinical syndrome of AD in addition to the pathway from APP to Aβ plaque.

### 5.3. RNA Therapeutic Target

Controlling and removing Aβ from the brain is known to protect neurons against Aβ-induced pathology, since it has been linked to the beginning of AD and cerebral amyloid angiopathy [120]. APP and Aβ have been proposed to have a variety of innate activities, many of which support nervous system development and function [121]. Instead of just inhibiting Aβ synthesis and/or extracellular export in the first place, primates have developed a variety of strategies and put forth a significant amount of effort to remove it from the brain [120]. Therefore, it is probable that this clearance is the beneficial function in and of itself. Aβ clearance has an adaptive purpose in addition to preventing its pathological effects. In preclinical and early phase trials, therapeutic attempts to eliminate or reduce the generation of Aβ were promising; but, in later phases of clinical trials, these strategies have mainly failed to change the course of AD [122].

The failure of the amyloid-targeting pharmacological trials has mostly been attributed to a lack of sufficient specificity and accurate translational models, loss of Aβ physiological homeostasis, and difficulty to administer at the ideal therapeutic window. AD is being treated with drugs that target the 5′-UTR of the APP mRNA. This reduces the formation of harmful Aβ peptides by preventing the translation of APP mRNA. Selective mRNA targeting APP translation through the precursor transcripts uniquely folded 5′-UTR can decrease APP expressions by providing a novel target [13]. Using high throughput screening to find small molecules that inhibited translation controlled by APP 5′-UTR and demonstrated in vivo anti-amyloid efficacy with enhanced cognitive function [123]. It has been demonstrated that modulating Aβ aggregation with small molecules is a very successful therapeutic potential [124].

While 3-D structure is a more common pharmacological target for proteins, current RNA drug development depends on the secondary structure of RNA. RNA therapies have the advantage of having a smaller target size than proteins. The control of APP translation, which has been connected to the IRE signaling pathway, is what causes neurodegeneration in AD. Therefore, treating AD may benefit from the discovery of small molecule IRE-targeted inhibitors that reduce APP levels and Aβ aggregation. The cholinesterase inhibitor phenserine and its (+)-enantiomer posiphen, both decreased APP 5′-UTR mRNA dependent translation while reducing brain Aβ expression in vivo, are important examples of these small molecule compounds [125,126].

**Figure 4:**
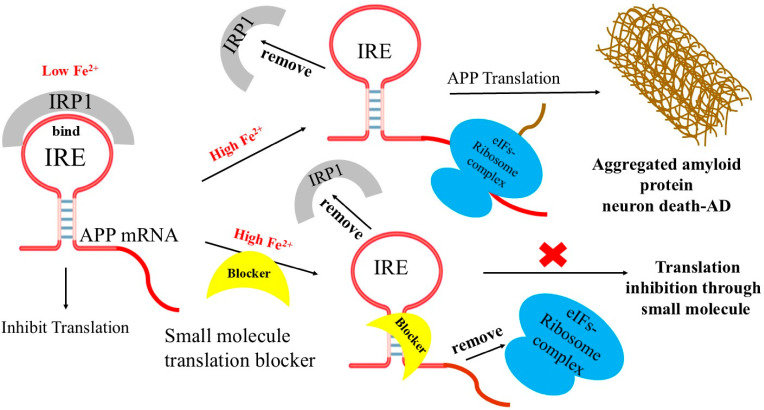
Model for the use of 5′-UTR APP IRE directed small molecule inhibitors therapeutic targets its mRNA by simple binding and selectively inhibiting amyloid translation. At low Fe^2+^ conditions, IRP1 binds APP IRE, inhibits amyloid translation. At high Fe^2+^ conditions, repressor (IRP1) dissociates and promotes the association of ribosome-initiation factors to APP IRE mRNA leading to overexpressed neurotoxic amyloid. Small molecule binds to APP IRE structure and selectively inhibits the amyloid translation through blocking of ribosome and initiation factor binding to APP mRNA scanning sites.

Figure 4 proposed model describes a link between IRP, APP mRNA, and iron-dependent translation of amyloid expression. Repressor protein IRP1 binds to APP IRE mRNA at low cellular iron levels, preventing ribosome-initiation factors complex binding to regulate the production of neurotoxic amyloid protein. However, when cellular iron levels are high, IRP1 repressor releases from APP mRNA and encourages the assembly of activator (ribosome-initiation factors) binding to APP mRNA. This results in the overexpression of neurotoxic amyloid protein, which causes amyloid aggregation, fibrillation, and the advancement of AD. The current target of RNA-based anti-amyloid therapy is the APP IRE mRNA. Selective RNA targeting APP translation via the distinctively folded 5′-UTR of the APP mRNA transcript by small molecules can suppress this APP expression.

A model of APP IRE mRNA (Figure 4) illustrates examples of small molecule-regulated IRE-mRNA/IRP inhibition of protein synthesis, where ribosome binding is prevented. These compounds intercalate into the APP RNA 5′-UTR, and able to form hydrogen bonds with the phosphate backbone of RNA helix [127]. At high brain iron level, by replacing IRP1 as a repressor binding partner with ribosome initiation factor complex as an activator of protein overexpression to the APP IRE stem loop, small molecules could directly block the binding and scanning of ribosome-initiation factor complex on the APP 5′-UTR mRNA and inhibit translation. One of the well-characterized APP 5′-UTR-directed small molecule is posiphen, was found to block translation of APP and limit amyloid levels, both in neural cell lines and in mice model [125,128]. It functions as an APP translation blocker and is phenserine’s (+)-enantiomer. It has demonstrated anti-amyloid activity in patients after successfully completing Phase 1 clinical trials for AD. APP 5′-UTR is responsive to both IL-1 and metals, stimulates APP translation, and ultimately produces Aβ-peptide [63,129,130]. Based on this idea, SH-SY5Y transfected cells were used to screen a library of FDA-approved drugs and clinical trials [65,131,132]. The results were categorized as antibiotics, statins, metal chelators, mutagens, detergents, and blockers of receptor-ligand interactions that suppressed more than 95% of APP 5′-UTR translation [131,132]. N-acetyl cysteine (NAC), paroxetine, desferrioxamine (DFO), dimercaptopropanol, phenserine, and erythromycin were among those that inhibited the development of APP without changing the expression of amyloid-like proteins [132]. Additionally, without secretase activation, azithromycin inhibited the translation of luciferase reporter mRNA via the 146 nt APP 5′-UTR sequence [130]. When paroxetine and NAC were demonstrated to restrict amyloid levels in the TgCRND9 mice model of AD [123], the method of targeting APP 5′-UTR to lower amyloid expression was verified.

Increased Fe^2+^ levels during iron accumulation can destabilize the transferrin receptor and the divalent metal transporter mRNA and disrupt the binding of the IRE/IRP complex, which promotes the translation of ferritin and ferroportin transcripts [92]. Iron storage and export can therefore be increased, whereas Fe^2+^ absorption will be restricted under iron accumulation [93]. Iron homeostasis is compromised by pathological conditions that disrupt the IRP/IRE signaling pathway, which may be a contributing factor to the onset and progression of AD. In our earlier studies, we described a new mechanism for how iron affects target mRNA translation [133]. In vitro studies suggest that Fe^2+^ may bind to IRE stem-loop, potentially altering its conformation and modulating IRP affinity [133]. The interaction between the initiation factors-ribosome complex and IRE-mRNA will be facilitated by the conformational changes in mRNA brought on by iron binding, which can outcompete binding between IRP and the IRE mRNA [133]. Thus, up-regulated translation of target mRNA with a 5′-UTR IRE may be facilitated by iron buildup. The IRP/IRE signaling system may have physiological functions beyond maintaining iron homeostasis. Thus, APP IRE 5′-UTR translation blocker would anticipate inhibiting translation.

Table 1 summarizes representative small molecule therapeutics for AD that were targeted to the mRNA structures and demonstrated to limit expression of APP and Aβ levels. In TgCRND8 transgenic mice, dietary NAC reduced cortical Aβ levels have yielded “proof of principle” data for small molecule targeting of mRNA structures [123]. Posiphen and phenserine were demonstrated APP 5′-UTR directed translation blockers to reduced Aβ expression in vivo and enhanced cognition [134]. A group of benzimidazoles known as JTR-009 [135] are also similar to APP 5′-UTR blockers. As an intercalator, JTR-009 inhibited the translation of APP by preventing ribosome binding to the precursor transcript [13].

Several drugs have been found to interact with the APP 5′-UTR in previous studies, indicating the possibility of repurposing. These include the serotonin specific reuptake inhibitor (SSRI) antidepressant paroxetine, the macrolide antibiotic erythromycin, the antioxidant NAC, phenserine, carvedilol, yohimbine, and other intriguing small molecules that are presently being investigated in a number of human treatment trials for AD [136]. By focusing on the mRNA structure rather than the protein itself to control protein expression, these small molecules demonstrate a novel approach in AD treatment. The strategy is focused on the IRE of the 5′-UTR, which is crucial for regulating the synthesis of APP in response to cellular iron levels. By binding to a specific location in the IRE in solution, these molecules modify mRNA function [13,16]. These results showed that the RNA binding small molecules bind to folded RNA structures with the same selectivity in solutions as they do in living cells. Compared to other well-tolerated APP 5′-UTR directed translation blockers like posiphen, phenserine, JTR-009, and benzimidazole, were reported to restrict amyloid accumulation in mice models of AD by lowering the formation of toxic Aβ in SH-SY5Y neuronal cells [13]. These studies provide vital information for developing small molecules that selectively reduce the production of APP and aggregation of Aβ in AD brains. For the treatment of AD, these drugs that target the APP 5′-UTR offer a fresh way to discover RNA-based drugs that lower APP translation and Aβ-peptide synthesis.

Furthermore, it has been demonstrated that certain novel compounds such as paroxetine and DFO can prevent abnormal metal-promoted Aβ accumulation. A subset of these therapies produces the non-toxic sAPPα by modifying APP translation and cleavage via APP 5′-UTR dependent mechanisms. Their efficacy as genuine anti-amyloid drugs for the treatment of AD have been investigated in clinical trials [137,138]. Sjogren’s syndrome is treated with AF102B, a neuroprotective m-1 muscarinic agonist, in addition to these medications. Additionally, by activating the nonamyloidogenic pathway of APP expression (α-secretase), it can cause the release of APPs. This molecule capacity to enhance APP-Fpn complexes to stop the production of ROS and the efflux of toxically overloaded iron can also be investigated. Another type of therapeutic drugs that may be utilized to treat amyloid toxicity in AD are peptide-based small molecule inhibitors that target and modulate APP mRNA expression.

However, by taking lessons from this failure, we can better understand the process of APP progression and Aβ development of Alzheimer’s pathogenesis. Translational models and techniques that more precisely mimic the biology of AD are also required to reduce the gap between basic mechanistic research and clinical treatment. Most of the current AD treatments is symptomatic rather than curative. The AD treatments have advanced significantly over the past five years, especially with the advent of additional Aβ targeting monoclonal antibodies, in addition to the more popular cholinesterase inhibitors AChERs and NMDA.

**Table 1:** Small molecules targeted to the IRE structure in the 5′-UTR APP mRNA that inhibit APP translation and Aβ levels.

Compound Name	Structure	Action	APP Levels	Aβ Levels	Ref
JTR-009	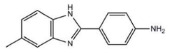	APP translation blockers	↓	↓	[13]
Phenserine/PS	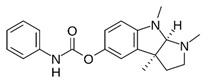	anticholinesterase	↓	↓	[139]
Paroxetine	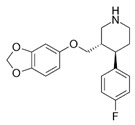	Serotonin reuptake inhibitor	↓	↓	[123,132]
Posiphen	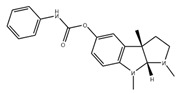	(+) enantiomer of PS	↓	↓	[75]
N-acetyl cysteine	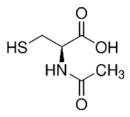	antioxidant	↓	↓	[123,134]
Erythromycin	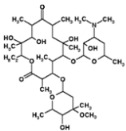	Macrolide antibiotic	↓	↓	[123]
Azithromycin	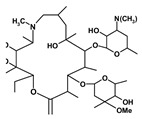	Macrolide antibiotic	↓	↓	[132]
Mycophenolic acid	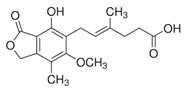	Immunosuppressant	↓	↓	[74]
Strophanthidine	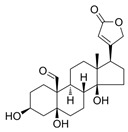	Plant glycoside	↓	↓	[74]
Desferrioxamine	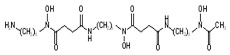	Fe chelator	↓	↓	[63]
Dimercaptopropanol	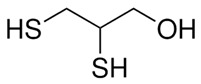	Hg chelator	↓	↓	[132]
Tetrathiomolybdate	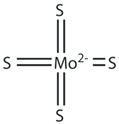	Cu chelator	↓	↓	[109]
Carvedilol	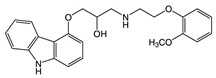	Aβ aggregation blocker	ND	↓	[136]

Arrows indicate decreased levels. ND, not determined.

## 6. Future Perspectives and Conclusions

These series of positive results offer fresh hope and indicate that targeting APP mRNA via the 5′-UTR with small molecule therapies may indeed be the right direction to follow. These small molecular drugs offer a potentially effective innovative approach to lowering Aβ levels and slowing the advancement of neurodegeneration in AD. We identified small molecule inhibitors that particularly interact with the 5′-UTR mRNA codes for the Alzheimer’s APP. APP 5′-UTR-directed inhibition of translation was reduced by a number of FDA-approved drugs. The pharmacological classifications include drugs, metal chelators, and bacterial antibiotics that recognized blockers of receptor-ligand interactions. These APP 5′-UTR-mRNA directed drugs represent a novel approach to reduce APP translation and Aβ peptide production for the treatment of AD. Due to insufficient precision regarding the pathophysiology of AD, the majority of Aβ targeting drug clinical trials have failed and yielded unsatisfactory outcomes. The increasing knowledge of the existence of an IRE stem loop structure in the APP 5′-UTR transcript function that iron dysregulation also plays a role in the pathophysiology of AD may lead to the establishment of a new hypothesis that will be crucial to future study.

RNA sequence-directed translation blockers that enhance brain iron homeostasis and decrease brain amyloid formation may eventually be evaluated clinically for AD treatment. In terms of the pathogenic side, new treatment approaches might include metal chelating drugs that target the IRE in the 5′-UTR mRNA, hence avoiding iron-induced toxicity and Aβ overexpression. By focusing on the functional IRE mRNA stem-loop that the APP 5′-UTR encodes, APP synthesis can be controlled. Iron chelating chemicals that specifically target the IRE in the 5′-UTR of the APP mRNA may be used in novel therapy techniques to prevent iron-induced toxicity, APP overproduction, and Aβ aggregation.

Future research aims to increase awareness of iron overload and support the development of RNA-based small molecule inhibitors to treat AD by elucidating the upstream and downstream roles of iron in AD pathogenesis, better understanding iron dysregulation, and revealing the function of APP mRNA as a post-transcriptional regulator of amyloid protein synthesis. In conclusion, focusing on the Aβ pathway targeting has opened a door that was closed for many years. Despite acknowledging that amyloid is just one component of a much larger complex mechanism, the field remains cautiously optimistic as it moves forward.

## Data Availability

No new data were created or analysed in this study. Data sharing is not applicable to this article.
